# Functional characterization of two rare BCR–FGFR1^+^ leukemias

**DOI:** 10.1101/mcs.a004838

**Published:** 2020-04

**Authors:** Evan J. Barnes, Jessica Leonard, Bruno C. Medeiros, Brian J. Druker, Cristina E. Tognon

**Affiliations:** 1 Division of Hematology and Medical Oncology, Knight Cancer Institute, Oregon Health & Science University, Portland, Oregon 97239, USA;; 2 Department of Medicine-Hematology, Stanford University, Stanford, California 94305, USA;; 3 Howard Hughes Medical Institute, Portland, Oregon 97239, USA

**Keywords:** acute myeloid leukemia, hematological neoplasm

## Abstract

8p11 myeloproliferative syndrome (EMS) represents a unique World Health Organization (WHO)-classified hematologic malignancy defined by translocations of the FGFR1 receptor. The syndrome is a myeloproliferative neoplasm characterized by eosinophilia and lymphadenopathy, with risk of progression to either acute myeloid leukemia (AML) or T- or B-lymphoblastic lymphoma/leukemia. Within the EMS subtype, translocations between *breakpoint cluster region* (*BCR*) and *fibroblast growth factor receptor 1* (*FGFR1*) have been shown to produce a dominant fusion protein that is notoriously resistant to tyrosine kinase inhibitors (TKIs). Here, we report two cases of *BCR–FGFR1*^+^ EMS identified via RNA sequencing (RNA-seq) and confirmed by fluorescence in situ hybridization (FISH). Sanger sequencing revealed that both cases harbored the exact same breakpoint. In the first case, the patient presented with AML-like disease, and in the second, the patient progressed to B-cell acute lymphoblastic leukemia (B-ALL). Additionally, we observed that that primary leukemia cells from Case 1 demonstrated sensitivity to the tyrosine kinase inhibitors ponatinib and dovitinib that can target FGFR1 kinase activity, whereas primary cells from Case 2 were resistant to both drugs. Taken together, these results suggest that some but not all BCR–FGFR1 fusion positive leukemias may respond to TKIs that target FGFR1 kinase activity.

## INTRODUCTION

8p11 myeloproliferative syndrome (EMS) is a rare syndrome characterized by the presence of a molecular disruption of the fibroblast growth factor receptor 1 (*FGFR1*) gene at the 8p11-12 chromosome locus, resulting in the formation of a novel fusion gene and subsequent production of a chimeric protein with constitutive activation of the FGFR1 tyrosine kinase (Jackson et al. 2010). In addition to the presence of this translocation, the syndrome is often characterized by, first, a myeloproliferative neoplasm usually associated with eosinophilia; second, lymphadenopathy usually associated with B- or T-cell acute lymphoblastic lymphoma/leukemia (T-ALL); and third, frequent progression to acute myeloid leukemia (AML) (Jackson et al. 2010; Arber et al. 2016). A small number of cases have been previously described as presenting with de novo B-cell acute lymphoblastic leukemia (B-ALL) or AML (Supplemental Table 1). In 2016, the World Health Organization (WHO) classified EMS as a myeloid/lymphoid neoplasm associated with eosinophilia, and the genes most commonly rearranged in these neoplasm include *PDGFRA*, *PDGFRB*, *PCM1-JAK2*, and *FGFR1* (Arber et al. 2016). 8p11 or *FGFR1* fusion positive EMS is distinct within this WHO category because of its relative resistance to tyrosine kinase inhibitors (TKIs) and poor prognosis (Arber et al. 2016). Less than 100 cases of EMS have been identified, and there is substantial heterogeneity in the partner genes of *FGFR1* translocations (Jackson et al. 2010).

**Figure 1.**
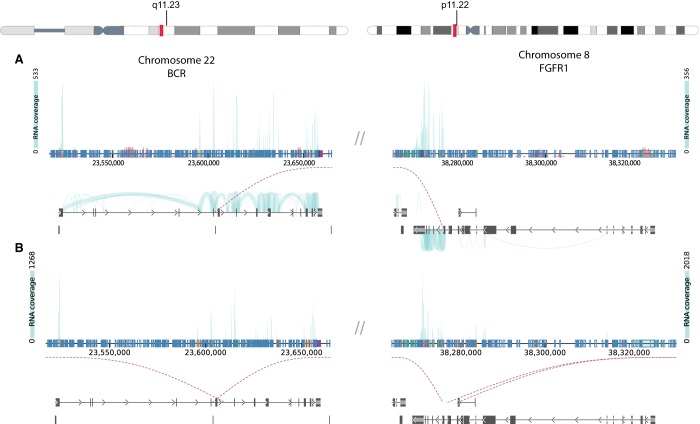
RNA sequencing (RNA-seq) identified potential BCR–FGFR1 fusions in two leukemia patient samples. DNA fusion report from the Vizome data visualization tool (www.vizome.org) were used to identify t(8,22) translocations corresponding to the *BCR–FGFR1* gene fusion. For Case 1 (*A*) and Case 2 (*B*), the *top* schematic illustrates the results of the RNA sequencing, which identified a probable *BCR–FGFR1* fusion. The height of the light blue plot illustrates the number of reads spanning each exon. Plots on the *bottom* illustrate the location of the translocation on each chromosome. Vertical blue and black bars in the *bottom* illustrations indicate exons of the respective gene, and the arrows indicate the direction of the forward reading frame. The purple dotted lines connect the two chromosomes together and identify the location of the suspected translocation. The chromosome schematics at the top of the figure identify the location of the potential translocation on the actual chromosome.

One rare *FGFR1* partner is the breakpoint cluster region (*BCR*) gene, leading to a t(8;22) translocation. To our knowledge, there have been 27 published cases of *BCR–FGFR1* EMS (Demiroglu et al. 2001; Fioretos et al. 2001; Pini et al. 2002; Murati et al. 2005; Ågerstam et al. 2007; Lee et al. 2008; Richebourg et al. 2008; Baldazzi et al. 2010; Patnaik et al. 2010; Kim et al. 2011; Wakim et al. 2011; Dolan et al. 2012; Haslam et al. 2012; Matikas et al. 2013; Morishige et al. 2013; Shimanuki et al. 2013; Khodadoust et al. 2016; Qin et al. 2016; Wang et al. 2016; Landberg et al. 2017; Montenegro-Garreaud et al. 2017; Liu and Meng 2018; Verstovsek et al. 2018; Villafuerte-Gutiérrez et al. 2018; Konishi et al. 2019). Unfortunately, most cases tend to be refractory to conventional induction chemotherapy and resistant to TKIs. Durable remissions have only occurred after allogenic stem cell transplant (ASCT) (Liu and Meng 2018; Konishi et al. 2019). The lack of an effective therapeutic strategy reduces treatment options for those ineligible for ASCT and limits the ability to bridge patients between diagnosis and transplantation. Using RNA sequencing (RNA-seq), we identified and confirmed *BCR–FGFR1* EMS in two patients, one presenting initially as AML (Fig. 1A) and the other as B-ALL (Fig. 1B). Further, drug sensitivity tests performed on both cases showed that cells from the AML patient sample exhibited sensitivity to ponatinib and dovitinib, whereas the B-ALL patient sample cells were resistant to these two same drugs.

## RESULTS

### Case Presentations

#### Case 1: BCR–FGFR1^+^ AML

**Figure 2.**
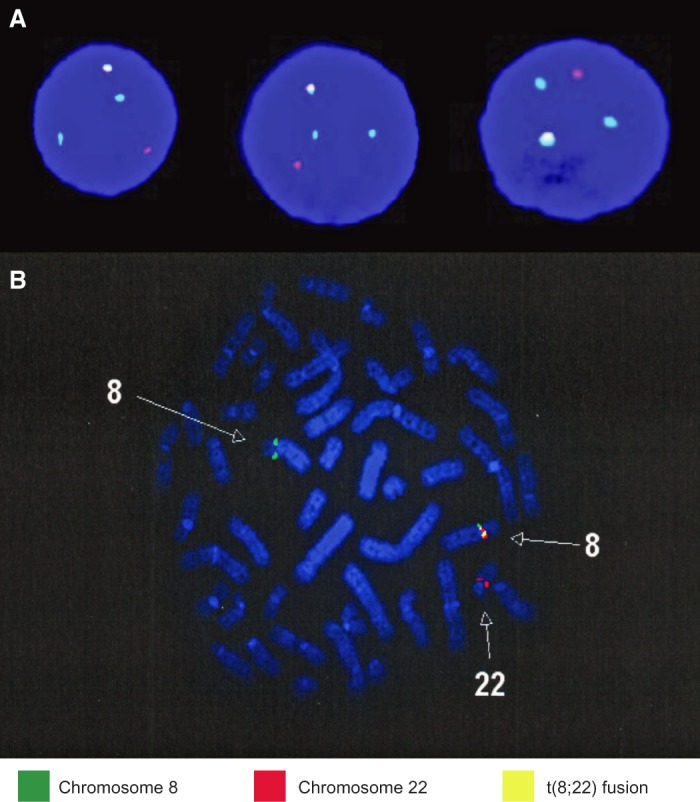
Fluorescence in situ hybridization (FISH) panel. The FISH panel results identify the presence of the t(8;22) translocation in both patients. Two hundred cells were analyzed for disruption in *FGFR1* using *FGFR1* flanking probes, and cases were considered positive if >15% of cells displayed split signals. The Case 1 FISH panels (*A*) were analyzed using *FGFR1* separation probe (Cytocell), and the Case 2 FISH panel (*B*) was performed using a *FGFR1* break-apart probe (Poseidon). Both panels demonstrated der(8) and der(22) along with fusion t(8;22).

The first case is a 58-yr-old man who reported generalized weakness and night sweats for 1 week after initial complaints of dyspepsia, abdominal distention, and early satiety. Laboratory studies indicated a leukocytosis (150,000/µL) with 72% blasts and mild absolute basophilia, anemia (Hgb 7.1 g/dL), and thrombocytopenia (Plt 88K/µL), which was concerning for de novo AML. As the patient's hyperkalemia (5.8 mg/dL) and creatinine levels (1.14 mg/dL) were concerning for tumor lysis syndrome, the patient was emergently transferred and started on aggressive IV fluid replacement therapy, allopurinol 300 mg twice daily, and 2000 mg hydroxyurea. The bone marrow biopsy was found to be hypercellular for age (>90%) with diffuse sheets of blasts (57%). Background trilineage hematopoiesis was markedly decreased, and erythroid cells were decreased in number with left-shifted maturation. The bone marrow aspirate was consistent with AML, and cytologic studies indicated an abnormality in *FGFR1*. Additional cytologic studies and fluorescence in situ hybridization (FISH) confirmation identified a t(8;22) clone, which was consistent with a *BCR–FGFR1* translocation (Fig. 2A). The patient's karyotype was recorded as 47, XY, t(8;22) (p11.2; q11.2), +19[20]. Genetic testing was positive for a RUNX1 mutation (p.S322fs*278) and two variants of PHF6 (p.G360R) and ATM (p.P604S). Genetic testing was negative for *NPM1*, *FLT3*, *CEBPa*, and *c-KIT* mutations.

Based on the result of the SORAML trial, the patient was started on sorafenib and 7 + 3 (Röllig et al. 2015). Complete remission with minimal residual disease (MRD) negative status was achieved after two induction cycles. Complete remission with MRD negative status was maintained until allogenic stem cell transplant. Despite transplant, disease relapse occurred, and FLAG-IDA (fludarabine, high-dose cytosine arabinoside, idarubicin, and granulocyte colony-stimulating factor) treatment was started. Shortly after commencing FLAG-IDA, the patient developed bacteremia and sepsis. The patient died shortly thereafter because of complications from septic shock.

**Figure 3.**
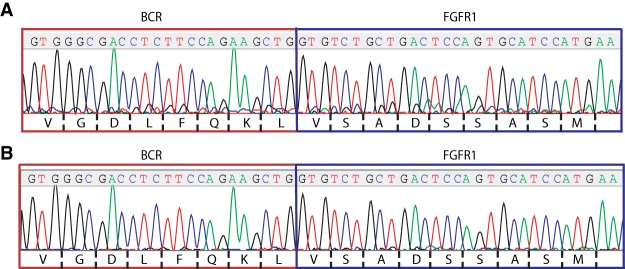
Sanger sequencing. Sanger sequencing identified the same breakpoint in the BCR–FGFR1 fusions found in both patients. cDNA was created from patient RNA samples via reverse transcription polymerase chain reaction (RT-PCR) using a *BCR* forward and *FGFR1* reverse primer. After purification, sequencing was performed with the same primers and compared to BCR–FGFR1 fusions described in a previous report (Landberg et al. 2017). Sanger sequencing trace files for Case 1 (*A*) and for Case 2 (*B*) demonstrate the same breakpoint, which matches the previous sequences reported in the literature.

**Figure 4.**
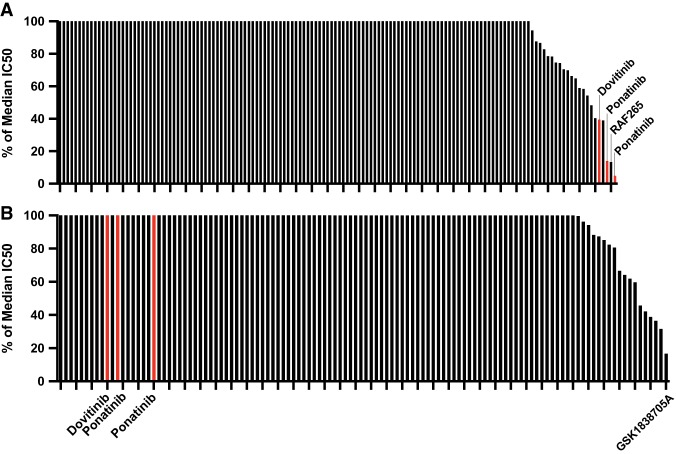
Case 1 samples demonstrate sensitivity to ponatinib and dovitinib, whereas Case 2 samples demonstrate resistance. Patient bone marrow cells were cultured for 3 d in gradients of small-molecule inhibitors to evaluate drug sensitivity patterns. The graphs show percent (%) of the median IC_50_ for cells from Case 1 (*A*) and Case 2 (*B*). Dovitinib and ponatinib results are highlighted in both cases. Additional sensitive inhibitors were also labeled in their respective cases. Drugs are considered highly sensitive for a given patient if they are < 20% of the median IC_50_, and in both cases, percent median IC_50_ values were capped at 100%. A full list of tested inhibitors and their drug sensitivity results is provided in Supplemental Table 2.

Upon initial identification of the *BCR–FGFR1* gene fusion via RNA-seq (see Table 2; Fig. 1A), the fusions were further confirmed in patient RNA samples using reverse transcription polymerase chain reaction (RT-PCR) and Sanger sequencing. Molecular analysis revealed the translocation with breakpoints occurring at residue L584 of BCR and V429 of FGFR1 (Fig. 3A). Ex vivo drug sensitivity testing was performed as previously described (Tyner et al. 2013) using cells freshly isolated from both samples to assess sensitivity to a panel of small-molecule inhibitors. Samples from Case 1 showed strong sensitivity to the TKIs ponatinib (% of the median IC_50_ = 4.869 and 14.025) and some sensitivity toward dovitinib (% of the median IC_50_ = 39.427) (Fig. 4A). Samples from Case 1 also showed sensitivity to the BRAF inhibitor RAF265 (% of the median IC_50_ = 13.349).

#### Case 2: BCR–FGFR1^+^ B-ALL

The second case is a 52-yr-old female who presented for evaluation after developing bruising, pruritus, low-grade fevers, night sweats, malaise, and abdominal fullness. A complete blood count was significant for a leukocytosis (40,000/µL), anemia (Hgb 8.4 g/dL, Hct 24%), and thrombocytopenia (Plt 21K/µL). A bone marrow biopsy supported a diagnosis of B-ALL with the immunophenotype CD10, CD19, CD20 (dim), CD34, CD79a, HLA DR, and TdT^+^, with a background of dyspoietic myeloid maturation supporting the evolution of EMS to B-ALL. Cytogenetic studies revealed a karyotype of 46 XX, t(8;22) (p11.2;q11.2), del(16) (q22) [9]/46, idem, del(7)(p13), del 9(p22) [5] (Fig. 2B). Genetic testing was negative for mutations in *ABL*, *AKT 1/2*, *CBL*, *CBLB*, *FBXW7*, *FLT3*, *FMS*, *GATA1*, *IDH2*, *JAK1/2*, *KRAS*, *MET*, *MPL*, *NRAS*, *NTRK1*, *PAX5*, and *SOS1*. The patient was started on HyperCVAD Part A and rituximab, but a bone marrow biopsy 14 d after starting treatment showed a hypercellular marrow (90%) with 50% residual disease with the immunophenotype CD10, CD19, CD22, CD34, CD79a, and TdT^+^, which was consistent with B-ALL. The patient was transitioned to HyperCVAD Part B, and postcycle bone marrow biopsy showed no evidence of residual disease despite persistence of a hypercellular marrow.

The patient then received three cycles of HyperCVAD Part B and a single cycle of high-dose methotrexate, but treatment was complicated by hepatotoxicity, resulting in a 2-mo chemotherapy hold. Before treatment could be resumed, the patient suffered a relapse with leukemic infiltration of the liver. The patient received a treatment cycle of FLAG-IDA, but treatment was complicated further by the development of invasive fungal pneumonia. The patient was admitted and treated for the fungal pneumonia. During admission, another bone marrow biopsy was obtained, which indicated persistent B-ALL (75% blasts in a 75% cellular marrow with the immunophenotype CD10, CD19, CD22, CD34, CD58, CD79a, and TdT^+^) with a background of erythroid and myeloid dysplasia. Her karyotype at progression was 46 XX t(8;22), showing persistence of only her *BCR–FGFR1* clone. Unfortunately, the patient was not a candidate for additional chemotherapy while she had the pneumonia. The patient died from her progressive leukemia 6 mo after initial presentation. A summary of the clinical characteristics for each case is provided in Table 1.

**Table 1.** Clinical case characteristics

Case	Age (years), sex	Clinical manifestation prior to EMS diagnosis	Karyotype	Treatments	Outcome
Case 1	58, male	AML	47, XY, t(8;22) (p11.2; q11.2), +19[20]	1. Sorafenib and 7 + 3 2. ASCT 3. FLAG-IDA	Originally, CR with MRD negative status Relapse 5 mo and death 6 mo after presentation
Case 2	72, female	B-ALL	46, XX, t(8;22), del(9), del(16), and del (7)	1. Hyper-CVAD-A and rituximab 2. Hyper-CVAD-B and methotrexate 3. FLAG-IDA	Originally, CR with MRD negative status Death 6 mo after presentation

(CR) Complete remission, (MRD) minimal residual disease, (7 + 3) cytarabine (7 d) + anthracycline (3 d), (FLAG-IDA) fludarabine, cytarabine, idarubicin, and granulocyte colony-stimulating factor (G-CSF), (Hyper-CVAD-A) cyclophosphamide, vincristine, doxorubicin, and dexamethasone, (Hyper-CVAD-B) methotrexate and cytarabine.

Upon initial identification of the *BCR–FGFR1* gene fusion via RNA-seq (Table 2; Fig. 1B), the fusion was further confirmed in the patient's RNA samples using RT-PCR and Sanger sequencing. As in Case 1, molecular analysis revealed a translocation between BCR–FGFR1 cases with breakpoints occurring at residue L584 of BCR and V429 of FGFR1 (Fig. 3B). Ex-vivo drug sensitivity testing was performed as previously described (Tyner et al. 2013) using cells freshly isolated from both samples to assess sensitivity to a panel of small-molecule inhibitors. Samples from Case 2 exhibited resistance to dovitinib and ponatinib, with a percent of the median IC_50_ values greater than 100% (Fig. 4B). Interestingly, the sample from Case 2 was sensitive to the insulin-like growth factor-1 receptor inhibitor GSK1838705A (% of the median IC_50_ = 16.685). The Case 2 samples were highly resistant to most drugs.

**Table 2.** BCR–FGFR1 fusions detected by RNA-seq

Case #	Left gene	Left chromosome	Left position	Right gene	Right chromosome	Right position	# of spanning mate pairs	# of mate pairs one end only spanning	Strand info	Left flanking sequence	Right flanking sequence
1	*BCR*	8	38275890	*FGFR1*	22	23603726	9	45	fr	TGCAGCAGTGG AGCCACCAG CAGCGGGTG GGCGACCTCTT CCAGAAGCTG	GTGTCTGCTGA CTCCAGTGCA TCCATGAACT CTGGGGTTC TTCTGGTTCG
2	*BCR*	8	38275891	*FGFR1*	22	23603727	107	15	fr	GTGCAGCAGTG GAGCCACCAG CAGCGGGTGG GCGACCTCTTC CAGAAGCTG	GTGTCTGCTGAC TCCAGTGCATC CATGAACTCTG GGGTTCTTCTG GTTCGG

## DISCUSSION

Molecular and cytogenetic analysis in both cases revealed several interesting areas for analysis. In both cases, the same translocation was noted, with breakpoints occurring at residue L584 of BCR and V429 of FGFR1 (Fig. 3A,B). This same BCR–FGFR1 translocation pattern was previously reported, with exons 1–4 of *BCR*, containing the coil–coil domain, fused with exons 9–17 of *FGFR1*, containing the tyrosine kinase domain (Khodadoust et al. 2016; Landberg et al. 2017). Cytogenetic analyses in Case 1 involved the *BCR–FGFR1* translocation and trisomy 19, and Case 2 involved the *BCR–FGFR1* translocation along with deletions in Chromosomes 7, 9, and 16. Only del 16 was found to be in the same clone as the t(8;22), and this deletion subsequently disappeared at progression. Interestingly, trisomy 19 is a known chromosomal abnormality seen in EMS as it transforms to acute leukemia (Jackson et al. 2010). It has been suggested that the additional molecular abnormalities may drive acute transformation and play a role in the pathogenesis, but little specific evidence exists at this time (Montenegro-Garreaud et al. 2017). Many cases of EMS do present with myeloproliferative neoplasm (MPN) prodrome, and the background of dyspoietic myeloid maturation in Case 2 is consistent with an original MDS that progressed to ALL. Interestingly, Case 1 is unusual in that the patient presented as de novo AML without any evidence of an MPN prodrome. Additionally, as indicated by the Flow Cytometry CD markers, Case 2 presented originally as B-ALL, as opposed to T-ALL. Although EMS is associated with both T- or B-ALL and AML, literature analysis suggests that B-ALL is relatively common in *BCR–FGFR1* fusions, making up around one-third of reported cases (Supplemental Table 1). This evidence suggests that the partner gene in *FGFR1* fusions may play a role in disease progression. The predominance of B-cell lineage transformation within *BCR–FGFR1*^+^ EMS may be related to the specific breakpoint within the *BCR* gene. Similar to what is observed in the minor form of BCR–ABL (m-bcr) in CML, The breakpoint in *BCR* exon 4 results in the loss of the RHOGEF/DBL and PH domains. This loss of the RHOGEF/DBL and PH domains is also observed in the p190 form of BCR–ABL (m-bcr; minor bcr) in CML which has been hypothesized to lead to a B-cell lineage blast phase (Montenegro-Garreaud et al. 2017).

Despite harboring the same BCR–FGFR1 breakpoint, samples from Case 1 and Case 2 showed surprising variation in TKI sensitivities. Samples from Case 1 showed strong sensitivity to the TKIs ponatinib (% of the median IC_50_ = 4.869 and 14.025) and some sensitivity toward dovitinib (% of the median IC_50_ = 39.427) (Fig. 4A). These results are consistent with previously reported sensitivity of dovitinib and ponatinib in *BCR–FGFR1* EMS cell lines (Landberg et al. 2017). Samples from Case 2, however, exhibited resistance to dovitinib and ponatinib, with percent of the median IC_50_ values greater than 100% (Fig. 4B). Although the cases presented with the same translocation, there is significant variation in both the original presentation (AML vs. B-ALL) and the likely pathogenic mechanisms contributing to acute transformation (+19 vs. del 7, 9, and 16). Because these factors would likely change the pathogenic drivers seen in the acute phase, it may account for the differing drug sensitivities (Table 1). Another possible explanation for the varying sensitivities relates to the difference between myeloid and lymphoid genealogy in samples. Previous studies have demonstrated sensitivity of ponatinib and dovitinib in BCR–FGFR1 fusion positive myeloid cell lines and myeloid lineage patient samples (Chase et al. 2007; Landberg et al. 2017), but their research did not extend into sensitivities in the lymphoid lineage. Thus, there is evidence to suggest that there may be differences in sensitivities to TKIs depending on whether patients present with myeloid versus lymphoid lineage disease, and further research should focus on identifying potential causes of this sensitivity discrepancy.

Interestingly, samples from Case 2 were sensitive to the insulin-like growth factor-1 receptor (IGF1R) inhibitor GSK1838705A (% of the median IC_50_ = 16.685). IGF1R is necessary for cell survival, and it is frequently overactivated in many malignancies. Previous research has suggested that IGF1R may play a critical role in several malignancies, particularly those with fusions (Werner et al. 2018; Andersson et al. 2019). One proposed mechanism is that the activation of many fusion proteins require transactivation of the *IGF1R* gene, which promotes receptor phosphorylation and aberrant fusion protein signaling (Werner et al. 2018). Several chromosomal fusions, such as the MYB-NFIB fusion in adenoid cystic carcinoma, show dependence on IGF1R signaling, and IGF1R shows promise as a clinical target (Xie et al. 2015; Andersson et al. 2019). Additionally, *IGF1R* is known to have a unique interaction with BCR–ABL. IGF1R plays a role in differentiation of hematopoietic cells and appears to regulate BCR–ABL leukemia cell fate and self-renewal in chronic myeloid leukemia (CML) cells (Xie et al. 2015). This interaction is mediated mainly through BCR, and it is possible that the BCR–FGFR1 may be targeted in a similar manner. Clinical trials with IGF1R inhibitors themselves have shown mixed results and many cases of resistance, but it is possible that *BCR–FGFR1* EMS cases may benefit from combined therapy with both TKIs and IGF1R inhibitors. Further research should explore the potential benefits of combined treatment.

Although they have additional targets, ponatinib and dovitinib are known to inhibit FGFR1 tyrosine kinase activity, which has prompted studies into efficacy against *BCR–FGFR1* cells before (Gozgit et al. 2012; Khodadoust et al. 2016; Landberg et al. 2017). The results here do indicate that inhibitors of FGFR1, like ponatinib and dovitinib, may be useful as early treatment for certain patients with *BCR–FGFR1* EMS until ASCT can be performed. Our evidence, however, does suggest that the effectiveness of the treatment may depend on the myeloid or lymphoid lineage in the leukemia, and those with myeloid lineage leukemia may be more sensitive to FGFR1 TKI inhibition. For certain patient populations, such as those with lymphoid progenitor-based malignancies, patients may be resistant to the drugs despite harboring the same BCR–FGFR1 protein. Considering the evidence in this report, it will be enlightening to see the results of the upcoming FIGHT-203 Trial (Clinical Trial #NCT03011372). This Phase 2 Open Label study aims to evaluate the effectiveness of the FGFR1-3 kinase inhibitor pemigatinib (INCB054828) in patients with myeloid or lymphoid neoplasm with FGFR fusions. Interim results of this trial reported on two identified *BCR–FGFR1* cases. One myeloid/lymphoid neoplasm case demonstrated complete response, but another lymphoid blast case demonstrated no response (Verstovsek et al. 2018). It will be useful to see how the effectiveness of FGFR inhibitors in treating additional FGFR fusion cases, and the data may reveal underlying genetic factors that make certain FGFR fusion neoplasms sensitive or resistant to FGFR inhibition.

In summary, these clinical cases represent, to our knowledge, the 28th and 29th reported cases of *BCR–FGFR1* EMS and the 16th and 17th cases of molecularly confirmed *BCR–FGFR1* EMS (confirmed via FISH and RT-PCR). Although they exhibit some similar clinical features, the cases show varying sensitivity to FGFR1 inhibitors like ponatinib and dovitinib. This report underscores the complexity of EMS treatment and the role that small-molecule inhibitors can play in bridging a patient to ASCT.

## METHODS

### Cytogenetics and Fluorescence In Situ Hybridization

Standard trypsin and Wright (GTW)-banded karyotype analysis was performed following standard clinical protocol and described following the international system for Human Cytogenetic Nomenclature (Arsham and Shaffer 2017). FISH techniques were performed following standard clinical protocols. In Case 1, 200 cells were analyzed for disruptions in *FGFR1*. Interphase nuclei were probed using *FGFR1* separation probe (Cytocell), which comprised two *FGFR1* flanking probes. One probe covered 272 kb on one side of the *FGFR1* gene, and the other covered 267 kb on the other side of the gene. In Case 2, 200 cells were analyzed for disruptions in *FGFR1*. Interphase nuclei were probed using a *FGFR1* break-apart probe (Poseidon), which comprised two *FGFR1* flanking probes. One probe covered 540 kbp containing the *FGFR1* gene, and the other served as a control probe binding the region near the centromere. Cases were considered positive when >15% of cells displayed split signals.

### Mutation Analysis

Mutation analysis was performed on the Case 1 patient sample using a panel of 76 commonly mutated genes in hematologic malignancies (GeneTrails Hematologic Malignancy 76 Gene Panel from Knight Diagnostic Laboratories). In Case 2, patient sample was analyzed using a panel of 31 commonly mutated genes in hematologic malignancies (Oncogene Panel from Knight Diagnostic Laboratories). A full list of tested genes is provided in Supplemental Table 3.

### RNA-seq Fusion Detection

RNA-seq was performed using methods previously described (Zhang et al. 2017). Samples were analyzed using the Agilent SureSelect Strand-Specific RNA Library Preparation Kit on the Bravo robot (Agilent) and sequenced on the HiSeq 2500 using a 100-cycle paired-end protocol. Gene assignments were created using Ensembl build 75 gene models on GRCh37. TopHat-Fusion (v2.0.14) was used to identify the gene fusion for Case 1 (Kim and Salzberg 2011), and the STAR-fusion algorithm was used to identify the gene fusion for Case 2 (Haas et al. 2017). The Vizome web application (www.vizome.org) was used to visualize data and identify fusions within the Beat AML data set (Tyner et al. 2018) and ALL patient samples.

### Sanger Sequencing

Sanger sequencing was performed on RNA isolated from the case samples to verify the translocations identified by RNA-seq. RNA was converted to cDNA through PCR amplification using Accuprime *Taq* DNA Polymerase System (#12339016, Thermo Fisher) and the following primers: BCRforward: 5′-GACGAGTCAGCAGATCGAGA-3′ FGFR1reverse: 5′-CCTGCTAGCATGGGAGTC-3′. PCR products were purified using Amicon 0.5 mL 30K Centrifugal filter (#UFC50306, Millipore). Sequencing was performed with the same primer. The sequence was compared to known *BCR–FGFR1* sequences identified in UniProt and previous *BCR–FGFR1* sequences described in the literature to verify breakpoint location (Khodadoust et al. 2016; Landberg et al. 2017).

### Small-Molecule Inhibitor Assay

Following informed consent, peripheral blood or bone marrow aspirate specimens were collected from patients with AML or ALL. Mononuclear cells were isolated from each specimen by Ficoll density gradient centrifugation and stored for subsequent use in experiments. Freshly isolated peripheral blood mononuclear cells (PBMCs) from the two patients were plated with a panel of small-molecule inhibitors arrayed in graded concentrations in 384-well cell culture plates. Cells were incubated with the drugs for 72 h, and cell viability was determined by a methanethiosulfonate (MTS)-based assay (CellTitre96 Aqueous One Solution, Promega) as described previously (Tyner et al. 2013). Briefly, cells incubated with MTS were read at 490 nm after 1–24 h using a BioTek Synergy 2 plate reader (BioTek). Cell viability was determined by comparing the treated cells to untreated controls. Cubic fit regression curves were created using the data, and IC_50_ values were calculated for each drug. Patient-specific IC_50_ values were used to calculate the percentage of the median IC_50_, and IC_50_ values were capped at 100%. Median IC_50_ values were calculated across all samples ever tested for a given drug. A full list of tested inhibitors and their drug sensitivity results is provided in Supplemental Table 2.

### Inhibitor Stocks

Kinase inhibitor stocks for ponatinib, dovitinib, and other small-molecule inhibitors in the screen were purchased from Selleck Pharmaceuticals, reconstituted at a concentration of 10 mM in dimethyl sulfoxide (DMSO), and then used to create threefold dilutions in a seven-point dose curve. Drugs were plated into 384-well plates containing media and stored at −80°C prior to use in each experiment.

## ADDITIONAL INFORMATION

### Data Deposition and Access

The interpreted fusion variants have been deposited in ClinVar (https://www.ncbi.nlm.nih.gov/clinvar/) under accession number SCV000994995.1. The aligned RNA-seq data set for Case 1 has been deposited at dbGAP (study: phs001657.v1.p1). The project page from GDC can be found at https://gdc.cancer.gov/about-data/publications/BEATAML1-0-COHORT-2018. The RNA-seq data for Case 2 has been submitted to dbGAP, accession number pending. The data is available upon request from the corresponding author (tognon@ohsu.edu).

### Ethics Statement

The study was approved by the Institutional Review Boards (IRBs) at Oregon Health & Science University and Stanford University. Samples were obtained with written, informed consent from all patients.

### Acknowledgments

The authors thank Beth Wilmot, Daniel Bottomly, and Shannon K. McWeeney for the RNA-seq fusion analysis. B.J.D. and C.E.T. and this work were supported in part by Howard Hughes Medical Institute. The results shown here are based on data generated by the Beat AML Program, a project supported in part by the Leukemia & Lymphoma Society and the Oregon Health and Science University (OHSU) Knight Cancer Institute and through National Cancer Institute (NCI) funding to B.J.D. (1R01CA214428). Beat AML acknowledges the AML patients and Academic Medical Center partners who contributed samples to this project.

### Author Contributions

E.J.B. performed PCR validation, created the figures, and wrote the manuscript. C.E.T. conceived of the project, contributed to experimental design/data analysis, and guided the writing of the manuscript. B.C.M. and J.L. provided clinical information on the patients and provided edits to the manuscript. B.J.D. performed a review of the manuscript and contributed funding for the project.

### Competing Interest Statement

B.J.D.’s potential competing interests are that he is on the Scientific Advisory Board of Aileron Therapeutics, ALLCRON, Cepheid, Vivid Biosciences, Celgene, RUNX1 Research Program, Gilead Sciences (inactive), Baxalta (inactive), and Monojul (inactive); he is on the Scientific Advisory Board of and has stock in Aptose Biosciences, Blueprint Medicines, Beta Cat, Third Coast Therapeutics, GRAIL (inactive), and CTI BioPharma (inactive); he was the scientific founder of MolecularMD (inactive, acquired by ICON); he is on the Board of Directors of and has stock in Amgen; he is on the Board of Directors of Burroughs Wellcome Fund and CureOne; he is on the Joint Steering Committee of Beat AML LLS; he has clinical trial funding from Novartis, Bristol-Myers Squibb, and Pfizer; and he has royalties from Patent 6958335 (Novartis exclusive license) and OHSU and Dana-Farber Cancer Institute (one Merck exclusive license). C.E.T.’s potential competing interests are that she is on the Scientific Advisory Board of Igynta Pharmaceuticals (inactive). J.L.’s potential competing interests are that she receives research funding from Amgen and is a Consultant for Takeda. B.C.M. has no conflicts of interest to declare. He is currently employed by Roche/Genentech. E.J.B. has no conflicts of interest to declare.

## Supplementary Material

Supplemental Material
