# Supplementary material for: Functional characterization of two rare BCR–FGFR1+ leukemias
Source: Cold Spring Harb Mol Case Stud. 2020 Apr;6(2):a004838. doi: 10.1101/mcs.a004838 (PMC7133745; doi:10.1101/mcs.a004838)
Supplement: Supplemental Material [file supp_6_2_a004838__index.html]

Functional characterization of two rare BCR–FGFR1+ leukemias — Supplemental Material 

# Functional characterization of two rare BCR–FGFR1+ leukemias

## Supplemental Material

- Supplemental\_Table\_1.pdf
- Supplemental\_Table\_2\_Sensitivity\_Data.xlsx
- Supplemental\_Table\_3.pdf
