# Supplementary material for: Functional characterization of two rare BCR–FGFR1+ leukemias
Source: Cold Spring Harb Mol Case Stud. 2020 Apr;6(2):a004838. doi: 10.1101/mcs.a004838 (PMC7133745; doi:10.1101/mcs.a004838)
Supplement: Supplemental Material [file supp_mcs.a004838_Supplemental_Table_1.pdf]

**Supplemental Table 1:** Previous Literature Reports of BCR-FGFR1 EMS

| Author, Year | Age (years), sex | Clinical Manifestation at time of Diagnosis | Chromosomal Abnormalities | FISH Confirmation | RT-PCR Confirmation |
|---|---|---|---|---|---|
| Fioretos, 2001 | 75, M | MPN | t(8;22) (p11.2;q11.2), t(9;21)(q34;q22) | Yes | Yes |
| Demiroglu, 2001 | 65, F | MPN | t(8;22) (p11.2;q11,2) | Yes | Yes |
| | 51, F | MPN | t(8;22) (p11.2;q11.2) | Yes | Yes |
| Pini, 2002 | 74, F | MPN | t(8;22) (p11.2;q11.2) | Yes | Yes |
| Murati, 2005 | 68, M | MPN/B-ALL | t(8;22) (p11.2;q11.2) | No | Yes |
| Agerstam, 2007 | 58, F | MPN | t(8;22) (p11.2;q11.2) | Yes | No |
| Lee, 2008 | 50, F | MPN/B-ALL | t(8;22) (p11.2;q11.2)[9]/idem,i(9)(q10)[11] | No | No |
| Richebourg, 2008 | 56, F | MPN | t(8;22) (p11.2;q11.2) | Yes | Yes |
| Patnaik, 2010 | 57, F | MPN | t(8;22) (p11.2;q11.2) | Yes | No |
| Baldazzi, 2010 | 70, F | MPN/B-ALL | t(8;22) (p11.2;q11.2)[10]/idem,del(3)(p11p21) del(7)(p12p15),add(8)(p23),-9[6] | Yes | Yes |
| Kim, 2011 | 59, M | Myeloid and T-cell Neoplasm | t(8;22) (p11.2;q11.2)+19[16] | Yes | Yes |
| Wakim, 2011 | 43, M | MPN/B-ALL | t(6 ;11)(q11 ;p13),-7,t(8;22)(p11.2 ;q11.2), del(9)(p13p22)[11] | Yes | No |
| Dolan, 2012 | 8, M | MPN | Inv(4)(p15.2q13)t(8;22;14)9p11.2;q11.2;q24)[19] | Yes | Yes |
| Haslam, 2012 | 21,M | MPN/B-ALL | t(8;22)(p12;q11)[8]/45,idem,der(3;9)(q10;q10), dic(7;11)(p11;q13),+r [cp3] | Yes | Yes |
| Morishige, 2012 | 50, M | MPN/T-cell Neoplasm | t(8;22) (p12;q11) | Yes | Yes |
| Shimanuki, 2013 | 58 F | B-ALL | t(8;22)(p11.2;q11.2)-16, add(19)(p13)[16] | No | Yes |
| Matikas, 2013 | 74, F | Undetermined | del(5)(q33q35)t(8;22)(p11.2q11.2) | Yes | No |
| Wang, 2016 | 56, F | B-ALL | t(8;22)(p11.2;q11.2)[9]/idem,+der(22)t(8;22)[10] | Yes | No |
| Khodadoust, 2016 | 47, M | MPN | t(8;22) (p11.2;q11,2)[10] | Yes | Yes |
| Landberg, 2017 | 21, M | CML | del(3)(q13q?26),der(8)t(3;8)(?q;?p11)t(3;22)(?q;?q11) der(22)t(8;22)(p11;q11) | Yes | Yes |
| Montenegro-Garreaud, 2017 | 41, F | MPN/B-ALL | t(8;22) (p11.2;q11,2)[7] | Yes | No |
| | 66, M | MPN/B-ALL | t(8;22) (p11.2;q11,2)[15] | Yes | No |
| Liu, 2018 | 41, M | CML | t(8;22) (p11;q11) | Yes | Yes |
| Villafuerte-Gutiérrez, 2018 | 66, M | CML | t(8;22) (p11;q11)[19] | Yes | No |
| Verstovsek, 2018 | 39, F | Myeloid-Lymphoid Neoplasm | t(8;22) (p11;q11) | Unknown | Unknown |
| | 68, F | Myeloid-Lymphoid Neoplasm | t(8;22) (p11;q11) | Unknown | Unknown |
| Konishi, 2019 | 48, M | MPN/B-ALL | t(8;22) (p11;q11) | Yes | Yes |
| Case 1, 2019 | 58, M | AML | t(8;22) (p11;q11) +19[20] | Yes | Yes |
| Case 2, 2019 | 72, F | B-ALL | t(8;22) (p11.2;q11.2), del(16) (q22)[9]/ idem, del(7)(p13), del 9(p22) [5] | Yes | Yes |

MPN=Mixed Phenotype Neoplasm; Idem=Cytogenetic findings seen in the first karyotype
