# Supplementary material for: Functional characterization of two rare BCR–FGFR1+ leukemias
Source: Cold Spring Harb Mol Case Stud. 2020 Apr;6(2):a004838. doi: 10.1101/mcs.a004838 (PMC7133745; doi:10.1101/mcs.a004838)
Supplement: Supplemental Material [file supp_mcs.a004838_Supplemental_Table_3.pdf]

Supplemental Table 3: Summary of genes tested in mutation panels

Case 1:<br>GeneTrails Hematologic Malignancies 76 Gene Panel (Knight Diagnostics)				Case 2:<br>Oncogene Panel (Knight Diagnostics)			
ABL1	ATM	BRAF	CALR	ABL	AKT1	AKT2	AKT3
CRLF2	CSF3R	FLT3	GNA13	BRAF	CBL	CBLB	FBXW7
HRAS	IL7R	JAK1	JAK2	FES	FGFR4	FLT3	FMS
JAK3	KIT	KRAS	MPL	GATA1	HRAS	IDH1	IDH2
MYD88	NOTCH1	NOTCH2	NRAS	JAK1	JAK2	JAK3	KIT
SOCS1	STAT3	PTPN11	ASXL1	KRAS	MET	MPL	NOTCH1
CREBBP	EP300	EZH2	KDM6A	NPM1	NRAS	NTRK1	PAX5
PTEN	SUZ12	DNMT3A	TET2	PDGFRB	PTPN11	SOS1	
IDH1	IDH2	BCOR	BCL6				
CBL	CBLB	CEBPA	ETV6				
FOXO1	GATA1	GATA2	ID3				
IKZF1	KMT2D	MEF2B	MYC				
PAX5	PRDM1	RUNX1	TCF3				
SRSF2	SF3B1	U2AF1	ZRSR2				
SMC1A	SMC3	STAG2	RAD21				
TP53	WT1	PHF6	BCL2				
BIRC3	CARD11	CD79B	FAM5C				
FBXW7	HNRNPK	NPM1	TNFAIP3				
TNFRSF14	SETBP1						

More Information about panels can be found at: https://knightdxlabs.ohsu.edu/
